# Participation of irradiated *Anopheles arabiensis* males in swarms following field release in Sudan

**DOI:** 10.1186/1475-2875-13-484

**Published:** 2014-12-11

**Authors:** Tellal B Ageep, David Damiens, Bashir Alsharif, Ayman Ahmed, Elwaleed HO Salih, Fayez TA Ahmed, Abdoulaye Diabaté, Rosemary S Lees, Jeremie RL Gilles, Badria B El Sayed

**Affiliations:** National Centre for Research, Tropical Medicine Research Institute, PO Box 1304, Khartoum, Sudan; Insect Pest Control Laboratory, Joint FAO/IAEA Division of Nuclear Techniques in Food and Agriculture, International Atomic Energy Agency, Wagramerstraße 5, PO Box 100A-1400, Vienna, Austria; Polo d’Innovazione Genomica, Genetica e Biologia S.C.a.R.L., Edificio D, 3 piano Polo Unico di Medicina ‘Santa Maria della Misericordia’, Loc. S. Andrea delle Fratte, 06132 Perugia, Italy; Institut de Recherche en Sciences de la Santé, BP 545 Bobo Dioulasso, Burkina Faso

**Keywords:** Malaria, Sterile insect technique, SIT, Mark release recapture, Dispersion, Survival, Population estimation

## Abstract

**Background:**

The success of the sterile insect technique (SIT) depends the release of large numbers of sterile males, which are able to compete for mates with the wild male population within the target area. Unfortunately, the processes of colonisation, mass production and irradiation may reduce the competitiveness of sterile males through genetic selection, loss of natural traits and somatic damage. In this context, the capacity of released sterile *Anopheles arabiensis* males to survive, disperse and participate in swarms at occurring at varying distances from the release site was studied using mark-release-recapture (MRR) techniques.

**Methods:**

In order to assess their participation in swarms, irradiated and marked laboratory-reared male mosquitoes were released 50, 100 or 200 m from the known site of a large swarm on three consecutive nights. Males were collected from this large swarm on subsequent nights. Over the three days a total of 8,100 males were released. Mean distance travelled (MDT), daily probability of survival and estimated population size were calculated from the recapture data. An effect of male age at the time of release on these parameters was observed.

**Results:**

Five per cent of the males released over three days were recaptured. In two-, three- and four-day-old males, MDT was 118, 178 and 170 m, and the daily survival probability 0.95, 0.90 and 0.75, respectively. From the recapture data on the first day following each release, the Lincoln index gives an estimation of 32,546 males in the natural population.

**Discussion:**

Sterile *An. arabiensis* males released into the field were able to find and participate in existing swarms, and possibly even initiate swarms. The survival probability decreased with the age of male on release but the swarm participation and the distance travelled by older males seemed higher than for younger males. The inclusion of a pre-release period may thus be beneficial to male competitiveness and increase the attractiveness of adult sexing techniques, such as blood spiking.

## Background

Despite a reduction in incidence since the late 1990s, malaria remains a major public health challenge to Sudan, causing between one and two million cases annually between 2010 and 2012 [1]. In view of this high burden of disease, the Tropical Medicine Research Institute in Sudan, with the support of the Food and Agriculture Organization of the United Nations (FAO) and the International Atomic Energy Agency (IAEA), initiated a study to assess the feasibility of integrating the sterile insect technique (SIT) as part of area-wide integrated pest management (AW-PM) to control *Anopheles arabiensis*, the main vector of malaria in Sudan, along the River Nile [2]. The SIT consists of sequential releases of large numbers of male insects [3] following the application of ionizing radiation to induce sterilization, aiming to result in the transfer of their sterile spermatozoids to wild, virgin females during mating. This induced sterility in the female population is expected to result in a progressive decrease in the pest population with each generation. The success of the SIT depends on sterile males being competitive with the wild male population within the target area [4, 5].

Competitiveness in the field is determined by the ability to survive, disperse and participate efficiently in natural mating behaviour. Unfortunately, the process of mass production in the laboratory under controlled conditions may modify the behaviour of sterile males compared to their wild counterparts, through genetic selection and subsequent loss of natural traits [6–10], and lead to a low level of competitiveness in the field [11, 12]. Reduced dispersal and survival [13], flight endurance [14] and restricted swarm site selection [15] have all been observed. Assortative mating behaviours could also take place in the field due to changes to the laboratory strain, even within a short period of colonization [16, 12]. Colonized *Culex tarsalis* males have been seen to swarm in different areas compared to wild ones [17] and to be discriminated against by wild females [18]. In addition to the rearing itself, the sterilizing radiation (even when optimized to balance the level of sterility induced and the competitiveness of the irradiated males) could also affect male quality: reduced emergence [19, 20], reduced longevity [19, 21, 22], and diminished sperm production [23] have all been observed in irradiated male anopheline mosquitoes.

In most culicine species, most notably *Anopheles*, *Culex* and *Ochlerotatus* species, mating is initiated in flight and so is associated with swarming behaviour of the males [24–26]. Charlwood and Jones [27] defined swarming as male behaviour associated with mating: a distinct time and place in which males and virgin females of the same species are brought into close proximity. Mating swarms have been observed in several *Anopheles* species including *Anopheles gambiae*, *Anopheles coluzzii* [28], *Anopheles melas* [29] and *An. arabiensis* [30]. The capacity of irradiated *An. arabiensis* males to participate in swarming, when released at different distances from the location of known swarm sites, was thus studied using mark-release-recapture (MRR) techniques [31–33].

In *An. arabiensis*, irradiation decreases the competitiveness of males as measured in semi-field cages [34] and the number of spermatozoa present in the testes of newly sexually mature males, and prevents further sperm production during their adult life [35, 23]. Little information is available, however, about the behaviour and mating ability of irradiated *An. arabiensis* in the field [36]. One study by Hassan *et al.* [33] observed the participation of irradiated *An. arabiensis* males in swarms by marking 950 irradiated males with fluorescent powder and before releasing them at a distance of about 50–80 m from known swarm sites; twenty-four marked individuals were recaptured over four days. The present study builds on these results, with releases of almost ten times as many marked males, of different ages, and from points at different distances and directions from the swarm sites, to further investigate the ability of released males to survive, disperse and locate swarms over a greater distance.

The ability of the laboratory reared males to disperse sufficiently to successfully mate is one of the fundamental quality parameters for the success of any control programme that includes an SIT component. Indeed, it is crucial to determine the mean distance that a sterile male is able to fly as these results will strongly influence the release strategy, defining the optimal distances between release points, in the case of ground releases, to effectively cover the total treatment area. Unfortunately, most of the traps used in the estimation of adult mosquito population, i.e., sticky ovitraps, visual traps, BG-Sentinel Traps and backpack aspirators fail to catch males efficiently [24, 37–39]. Observation of swarm participation could be a good alternative to the use of traps to study male dispersal.

The sexual competitiveness of sterile males could be also influenced by the age of males released into the field. Some studies suggest allowing males to emerge in mass-rearing facilities and keeping them for a few days to allow sexual maturation, sugar feeding and recovery from handling and irradiation to improve their competitiveness. Male mating performance and longevity are highly dependent on sugar feeding [40, 41]. Improvement in performance with age has also been observed by Oliva *et al*. [42] in *Aedes albopictus* and Krishnamurthy *et al.* [43] in *Culex quinquefasciatus*. Some MMR studies have also shown that survival may increase after a certain age in *An. gambiae* [44], *Anopheles culicifacies* [26], *Culex tritaeniorhynchus* [25], and *Aedes triseriatus* [45].

Moreover, in mosquito SIT programmes, only males will be released and efficient sex separation has to be achieved before release [46]. Among different suggested techniques, spiking blood meals with an insecticide or toxicant is one possible approach. In the SIT component of an *Anopheles albimanus* control programme in El Salvador, spiking blood with malathion allowed the elimination of more than 95% of females [47]. In *An. arabiensis*, an efficient technique using ivermectin has been demonstrated [34], though at least two days are required to ensure total elimination of females. The effect of male age on release (i.e., the number of days the adult males are held beforehand) will thus be investigated.

## Methods

### Identification of swarm sites

**Figure 1:**
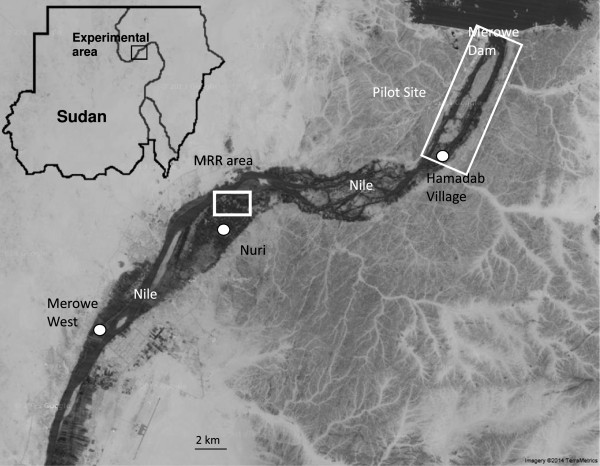
**The area of the Northern State in which an SIT feasibility pilot study is proposed.** In preparation for this pilot study *Anopheles arabiensis* swarms were surveyed at Merowe West, Hamadab Village, Nuri area and an abandoned and flooded brickworks southwest of Nuri in September 2012. In November 2012, swarms were only found in in Nuri and an MRR study was conducted to investigate participation of sterile males in swarms in the field.

**Figure 2:**
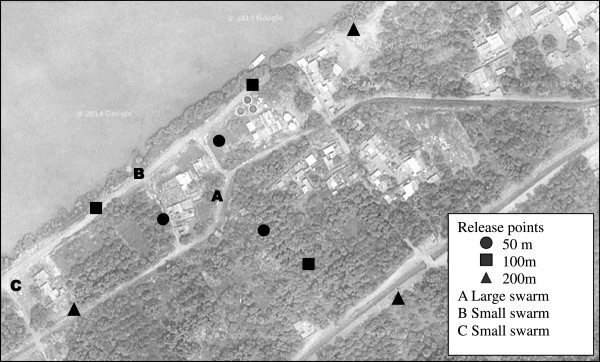
**Geographical layout of the mark-release-recapture study, conducted to investigate swarm participation in sterile irradiated** ***Anopheles arabiensis*** **males in Nuri, Sudan.** The letter A indicates the position of a large swarm, and B and C indicate two small swarms. The circles, squares and triangles indicate release points located 50, 100, 200 m from the site of the large swarm, respectively (Google maps).

The study was conducted in the Merowe area of Sudan, at a site 545 sq km in area along the banks of the Nile and containing 43 settlements (villages and semi-urban city residences), home to 51,444 inhabitants. This area was selected as it corresponds to the proposed release area for the pilot SIT feasibility study conducted by the Tropical Medicine Research Institute and FAO. Extensive surveys were conducted over approximately 45 man-hours in four locations (Figure 1) in September 2012 to identify swarming sitesin Merowe West, Hamadab Village (within the proposed pilot study area), Nuri area (south of the pilot site) and an abandoned but flooded brickworks southwest of Nuri. In November 2012, in preparation for the MRR, swarms were found in only the Nuri area where releases were thus performed (Figure 2).

### Description of the area used for the mark-release-recapture (MRR) study

The Nuri area of Sudan is abundantly forested with date and mango trees. Open fields are less common than elsewhere in the pilot study area. This area is also deeply penetrated by irrigation canals and is one of the broader areas of cultivated land along this stretch of the Nile riverbank in the vicinity of Merowe.

### Production of irradiated males

*Anopheles arabiensis* (Dongola strain, originating from Sudan) was cultured at the Tropical Medicine Research Institute laboratory (Soba, Khartoum, Sudan) following the anopheline-rearing procedure described in Balestrino *et al.* [48]. *Anopheles arabiensis* pupae were sexed under a stereomicroscope by observing the shape of their terminalia [49]. Male pupae were irradiated at 22–26 hours old using a Cobalt-60 Gammacell (Nordion 220) at the Soba laboratory of the Sudan Atomic Energy Commission (SAEC) with a sterilizing dose of 70 Gy [50].

### Transportation to the MRR site

One day after emergence, mosquitoes were transported from Khartoum to Merowe in batches of 75 adult males placed in paper cups and provided with sugar solution. The cups were placed in a cardboard box and wetted towels placed on top of the cups to maintain high humidity. Transportation was by road with a project vehicle and took four hours; this treatment has previously been shown to result in minimal mortality [50].

### Marking

At Merowe, adult males were marked with fluorescent dust (RADGLO® JST, Radiant NV, Houthalen, Belgium) in a paper cup covered with netting (75 males per cup) one hour before release. A 12-ml syringe fitted with a needle (Ava-med SD) was used to deliver a cloud of fluorescent dust under pressure into each cup in a sufficient quantity to dust all males. Nine colours were used, one colour for each of the three release distances (the same colour was used for each of the three release points at each distance) on each of the three release days.

### Male releases

In order to assess their participation in swarms, laboratory-reared, irradiated and marked male mosquitoes were released 50, 100 or 200 m from the known site of a large swarm, and this and two smaller swarms found nearby were sampled to determine composition (marked and unmarked males). Figure 2 shows the geographical layout of the experiment. Release points and swarm positions were georeferenced using a global positioning system Garmin 12XL [51] and Arc GIS software [52]. Releases were conducted at three points for each distance. Each day for three days, 900 males were released per distance (300 per point) at 17.00 hours (75 min before the beginning of the swarm). Unfortunately, due to logistical problems, the first release was conducted just after sunset at 19.00, and the collection on the first day was cancelled. In total, over the three days, 8,100 males were released. Coming from the same cohort, but released over three days, males varied in age according to the day of release (two, three or four days post-emergence for days 1, 2 and 3 of release, respectively). The effect of male age at release on swarm participation, dispersal and survival was assessed.

### Recapture

Mosquitoes were recaptured from three swarms (Figure 1): the large central swarm and two smaller swarms. Observations were made by looking at the sky in the direction of the lightest areas, 1–4 m above the ground. The swarms were sampled every day at 18.15 (during dusk) by attempting to collect all males swarming at each site using an aerial net. The sampling effort was comparable at each swarm and between the five days (using the same personnel, materials, and duration of time spent catching). All collected individuals were then anaesthetised in a freezer, examined to identify species, and observed under black light to identify marked males, and all numbers recorded. The recapture effort was limited to three days after the release because the objective was not to estimate long term survivorship or maximum flight range of released males, but rather movement from potential breeding sites into upland congregation sites.

### Data analysis

For all parameters, a global estimation was first made for the totality of males recaptured and then the effect of age on these parameters was studied by repeating the calculation with the males recaptured from each release day separately. Three groups of males were thus analysed, two-, three- and four-day-old males from release days 1, 2 and 3, respectively.

**Figure 3:**
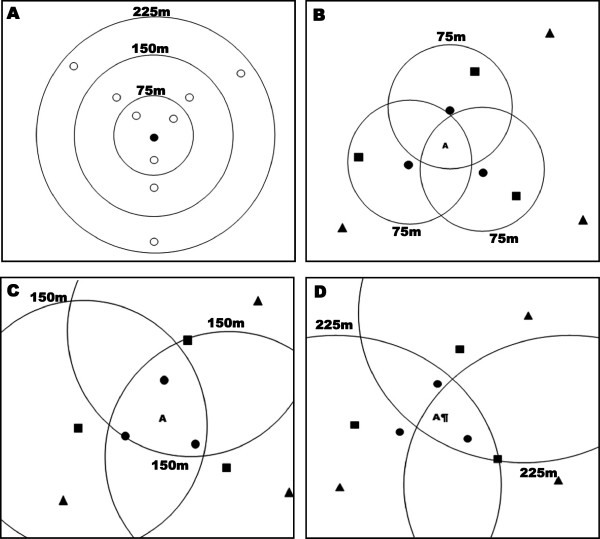
**Release-recapture setup for calculation of MDT. Annuli (75, 150, 225 m) are used to calculate MDT. A** represents the classical MDT set up. The black circle indicates the release point and white points indicate the recapture points. **B, C and D** represent the actual release set up in Sudan (see Figure 2) and the representation of the annuli for the modified MDT calculation. **B** represents the three annuli 75 m from the three release points and the letter **A** represents the large swarm, considered as one recapture point for each release point. The circles, squares, and triangles indicate release points located 50, 100, 200 m from the large swarm site, respectively.



Recapture rates were calculated as a proportion of the total number of marked mosquitoes recaptured divided by the total number originally marked and released. Dispersal of the released males was calculated as the mean distance travelled (MDT) [53], with compensation for unequal trap densities within each annulus [54]. In the classical MDT calculation, annuli separated by a chosen distance, 75 m in this experiment, are drawn around the release points, ensuring there is at least one trap in each area, and MDT can be estimated according to the recaptures in those traps (Figure 3A) [53].

Median distance of annulus is (inner radius + outer radius)/2 and ER is the number of recaptures that would be expected if trap density was equal in each annulus:


CF is a correction factor to account for differences in trap densities among annuli:


However in this study, the MDT calculation has been adapted since the experiment consisted not of one release point surrounded by several traps but instead only one ‘trap’ (the large swarm) and several release points surrounding it at various distances. Thus, each release point has been considered as one release point with one trap (large swarm) at a certain distance. For example, for releases at 50 m from the large swarm, from each release point there is one trap at 50 m. Since there were three release points, the large swarm can be described as three traps corresponding to the three release points. So for calculation of MDT, there are three traps at the distance of 50 m (Figure 3B). The same applies for the two other distances: 100 m (Figure 3C) and 200 m (Figure 3D), meaning that in total, data have been collected from the equivalent of nine traps, three for each distance. If three annuli are considered (0–75, 75–150, 150–225 m), MDT can be estimated. To estimate MDT according to male age, only the ER of the release day has been used for the calculation, i.e., ER1, ER2 and ER3 for two-, three- and four-day-old males, respectively. To avoid biases, the two small swarms were not used for dispersal evaluation because they were very close to the release points (Figure 2).

The daily survival probability was calculated by regressing the total number of males recaptured per day, transformed by log (x + 1), from the large swarm (trap) for the three release days. The daily survival probability was also calculated according to the age of males on release by regressing the number of males recaptured per day originating from each release day. Survival probability was estimated from the result of the antilog of the slope of the regression line [55, 56]. The effect of male age on survival was estimated by comparing the slopes of the three regression lines (T-test).

Population size was estimated only for the first day of recapture from the large swarm following each release (i.e., the first three days of recapture). The Lincoln index was modified for low recapture rate [24] as P = [as^t^ (n-r + l)]/(r + 1), where P is the estimated population density, a = the number of marked males released, s = the estimated probability of daily survival, t = the sampling day post-release, n = the total number of marked and unmarked males captured (since collections were made from swarms we can assume that most of the mosquitoes captured were males) and r = the number of marked males recaptured on the first day.

Distance was not taken into account when calculating either daily survival or population size, and the number of mosquitoes recaptured at different distances was pooled.

## Results

### Identification of swarm sites

In contrast to swarm surveys conducted in Merowe and Hamadab village, where no swarms were observed, nearly 30 swarms of *An. arabiensis* were detected in the Nuri area in September 2012. A preliminary larval survey in the Nuri area indicated the presence of both culicine and anopheline larvae in a breeding site in the water station of Nuri. No quantitative measurement of larval density was taken but qualitative observations indicated that larval density was higher in Nuri than in either Hamadab or Merowe. From the 28 swarms observed in the Nuri area, 15 were observed over 4 four days and were seen at the same sites each day. From the swarms found in September, three swarms were still detectable in November when the MRR experiments were conducted: a large swarm (18°34’21” N 31°53’08” E) and two smaller swarms (swarm A 18°34’23” N 31°53’07” E and swarm B 18°34’19” N 31°53’01” E) (Figure 2).

### Weather and climate

During the study period (23 to 28 November 2012), the Regional Meteorological Centre in Karima (3 km from the study site) reported a mean daily temperature of 29.6 ± 2.0°C. The variation was mainly due to the low temperature on the first day (26°C), with the following days ranging between 30 and 31°C. During the three release days, the wind direction was south to north with a speed of 12, 5 and 10 km/hour on each day.

### MRR data

Swarms were observed at all of the three sites on each of the five evenings of recapture. Observations are in accordance with the descriptions of Hassan *et al*. [33]. Swarming was at dusk, correlating with the time of sunset, which took place around 18:30. The height of the swarm above the ground, measured from its bottom edge, ranged from 1.5 to 2.5 m. The height of the swarm itself did not exceed 1.5 m.

A total of 1,140 *An. arabiensis* adults were captured from the three swarms over the five days of collection. Of the 8,100 male released over three days, a total of 442 (5.5%) were recaptured. According to the release day (age of male), the recapture rates were 1.08, 1.86 and 2.75% for two-, three- and four-day-old males, respectively.

### Large swarm

Marked male mosquitoes were recaptured from the large swarm on every evening following the releases (Table 1). On average, 235.0 ± 58.2 (mean ± standard error) mosquitoes were collected per night with a peak of 310 on the third day, the day of the third and final release, and the lowest catch of 168 on the fifth and last day. Over five collections, the proportion of marked males found in the large swarm varied between 17.2 and 42.3% (mean of 31.8 ± 11.4%). From the day the first males were released, a mean of 2.3 ± 0.2, 4.4 ± 3.2, 3.4 ± 1.1, 2.1 ± 0.5 and 1.4% marked males were collected on days 1 to 5, respectively. Males collected on the fifth day included some released on the first day, demonstrating that some males were able to survive for at least five days in the field. For each of the three days following each release, the proportion of marked males caught decreased with the distance from release. From the 900 males (300 per distance) released per replicate, the total proportion of marked males captured from the large swarm originating from releases over three days at 50, 100 and 200 m were 4.8 ± 2.1, 2.6 ± 2.4 and 1.8 ± 1.6%, respectively.

**Table 1:** ***Anopheles arabiensis*** **mosquitoes (male and female) collected from the large swarm in Nuri, in the Merowe region of Sudan, following three consecutive days of release of marked male adults**

Collection		Marked males								
		Released on day 1			Released on day 2			Released on day 3		
	Total number	50 m	100 m	200 m	50 m	100 m	200 m	50 m	100 m	200 m
Day 1	-	-	-	-	-	-	-	-	-	-
Day 2	227	7	13	0	17	0	2	-	-	-
Day 3	235	15	4	0	1	2	23	16	5	1
Day 4	310	14	8	0	33	4	0	26	29	17
Day 5	168	7	6	0	16	0	0	16	14	7

Released males were marked with nine different colours of fluorescent dust, such that males released from 50, 100 or 200 m from the location of the largest swarm on each of three consecutive days of release could be distinguished.

The modified MDT was calculated according to the recaptures from the large swarm (the ‘trap’) according to the distance from the release points (Table 2) to give a result of 162 m. When male age was taken into account, the MDT of two-, three- and four-day-old males is 89, 133 and 128 m, respectively.

**Figure 4:**
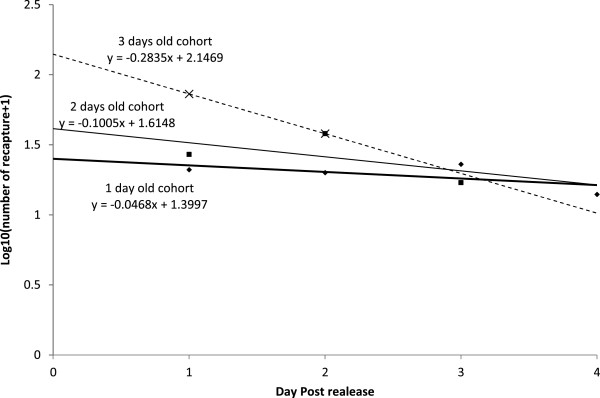
**Regression lines of recaptures (expressed as log number of released males recaptured + 1) of cohorts of** ***Anopheles arabiensis*** **released at two, three and four days of age.** The antilog of the slopes of regressions lines gives the daily survival probability.

The slope (−0.317) of the regression line for adult recaptures yielded a daily survival probability of 0.73. Regression lines were also estimated for the cohort of each release day to determine the survival probability by age of male on release (Figure 4). The slope of two-day-old males is not different from the slope for three-day-old males (t-Test, t = 0.24, df = 3, P = 0.83) but significantly different from the slope for four-day-old males (t-Test, t = 3.25, df = 4, P < 0.05). The slope of the three-day-old males is not significantly different from the slope for the four-day-old males (t-Test, t = 1.27, df = 3, P = 0.29). For the two-, three- and four-day-old males, the daily survival probabilities were 0.95, 0.90 and 0.75, respectively.

**Table 2:** **Step-by-step calculation of mean distance travelled (MDT) of** ***Anopheles arabiensis*** **males**

	Annulus			
	1	2	3	
A. Radius inner (km)	**0**	**0.075**	**0.150**	
B. Radius outer (km)	**0.075**	**0.150**	**0.225**	
C. Area (km)	**0.017672**	**0.053015**	**0.088358**	
D. Area total				**0.159044**
E. Number of trap	**3**	**3**	**3**	
F. Total of traps	**9**			
G. CF = (C/D) × F	**1**	**3**	**5**	
H1	**43**	**31**	**0**	
I1. ER1 = (H1/E) × G	**14.333**	**31**	**0**	
H2	**67**	**6**	**25**	
I2. ER2 = (H2/E) × G	**22.333**	**6**	**41.666**	
H3	**58**	**48**	**25**	
I3. ER3 = (H3/E) × G	**19.333**	**48**	**41.666**	
J. Sum of ER	**56**	**85**	**83.333**	
K				**224.333**
L. Distance (A + B)/2	**0.05**	**0.15**	**0.25**	
M.	**2.8**	**12.75**	**20.833**	
N				**36.383**

A: inner and B: outer radii of each annulus are recorded as A and B, C: area of each annulus, D: total area of the annuli, E: number of recapture sites in each annulus, F: the total number of traps, G: correction factor for each annulus, Hn: number of *An. arabiensis* recaptured in each annulus for the release day n, In: ERn estimated recaptures for the release day n, J: sum of ERn for each annulus, K: annuli sums totalled, L: median distance of each annulus, M: median distance (L) multiplied by its respective J, N: sum of M. MDT is calculated by the formula N/K.

From the total recapture data on the first day following each of the three releases, the Lincoln index gives an estimation of 32,546 males in the natural population. Variation in the Lincoln index was observed according to which release day (corresponding to male age) the data was used from: the population of males was estimated to be 25,400, 18,900 or 6,630 individuals when calculated from the data for two-, three- and four-day-old male releases, respectively.

### Small swarms

On average, a mean of 40.0 ± 220.0 and 19.2 ± 9.9 mosquitoes (mean ± standard error) were collected per night from small swarms A and B, respectively. During the five evening collections, the proportion of marked males collected from the small swarms varied between 60.0 and 97.0% for swarm A and 26.7 and 54.5% for swarm B. The marked males found in the small swarms came mostly from the release of the same day (94.7 ± 7.4% and 88.9 ± 15.7%, from swarms A and B, respectively), while only 41.0 ± 10.9% of the marked males caught in the large swarm were released that same day. For swarm A, most mosquitoes were caught from the 50-m point releases, probably from the two nearby (Figure 1). In swarm B, most recaptured mosquitoes were caught from a 200-m point release, probably from the one nearby (Figure 1).

## Discussion

In the current study, irradiated *An. arabiensis* released into the field were able to find and participate in swarms, despite the effects of colonization, rearing and sterilization. Indeed, marked male mosquitoes were recaptured from swarms every evening following their release suggesting that they were consistently and successfully finding and participating in swarms, even on the day they were released. Observations of Helinski *et al.* [50], who demonstrated the ability of irradiated laboratory-reared males to participate effectively in swarms in a contained semi-field system, are thus confirmed in the field as also observed by Hassan *et al.* [33]. The participation of irradiated males in swarms on the evening of their release suggests that males are sexually active on release, as expected since the released males were all at least two days post-emergence. That irradiated males were even able to initiate swarms can be inferred from the observation that in one small swarm near a release point, 95% of the males present were marked individuals.

Survival of irradiated males post-release appeared not to be significantly impaired by their treatment prior to release. Released males were able to survive up to at least five days in the field, suggesting that they were able to find a sugar source, a requirement for males at the beginning of adult life to increase life span and reach sexual maturation [40, 41, 57]. Indeed, many *An. arabiensis* males will die in less than 48 hours without provision of sugar [58]. Moreover, the probability of daily survival (between 0.75 and 0.95 according to the age) was similar to the survival previously reported in wild *Anopheles* species, usually between 0.8 and 0.9 in the field [44, 59–62]. However, the probability of survival decreased with male age; males released at four days old died at a higher rate than younger males. Some impact of irradiation on male survival could have been expected, though results between studies are contradictory: an effect of irradiation on *An. arabiensis* male survival has been observed in small cages [63] while not in semi-field cages [64]. Reduced survival could also be a result of the effect of marking on longevity of males, and an alternative marking method (such as the use of a stable isotope given in the larval diet) should be used to check whether increased mortality is indeed due to powder marking or whether it results from rearing methods.

However long males are held before release, the survival probability appears to decrease with male age suggesting that keeping males in cages with sugar prior to release has no positive impact on survival. However, more males were recaptured from the release of four-day-old males, than from releases of younger males, suggesting that four-day-old virgin males participate more in swarming than their younger counterparts. But more than the pre-release period effect, age itself could be the main factor. Improvement in mating ability with age has been seen in *Ae. albopictus* where competitiveness of five-day-old males appears to be higher than in younger males [42]. In *Cx. quinquefasciatus*, 36–60 hour-old males from a sterile male-linked translocation strain are more competitive than 12–36 hour-old males [43]. Previous studies show that the peak of mating activity takes place between three and seven days for *Anopheles stephensi* [65] and *An. culicifacies* [66]. In *An. gambiae*, males younger than three days old achieved low rates of insemination [67] while higher rates occurred with seven-day-old males [68]. This could also explain why the MDT was higher for three and four-day-old males than two-day-old released males. Despite having no positive impact on survival, a pre-release period of three or four days may thus be beneficial for the efficiency of the SIT technique. Moreover such a pre-release period could be used to employ the sexing process using a blood meal spiked with ivermectin [34] which needs two or three days to be efficient.

The higher mating activity of older males could also explain the variation according to age in estimation of the population size of *An. arabiensis*. When all males are taken into account, the population of the study site is estimated to be around 32,500 males, but the estimates varied hugely when estimates were based on data separated by male age, ranging from 25,400 to 6,600 adults. Such estimates are known to be very variable between years, experiments and even recapture days within an experiment [69]. In our experiment, the fact that four-day-old males were recaptured at a higher frequency could explain the high variation of the population estimates since with the Lincoln index, the more released males that are recaptured compared to wild males, the lower the value of the population estimate.

The presence in the large swarm of irradiated males on the evening of their release from 200 m away suggests that either the position of the swarm or the swarm itself is attractive to male mosquitoes at a distance. Very little is known about how male mosquitoes aggregate or how females are attracted to the swarm [70]. Visual markers are thought to be important [71–73], and it has been suggested that both male and female *An. gambiae* generate semiochemicals (i.e., pheromones) which may be involved in mate finding [74]. Moreover, in this study, a MDT of around 100 m was estimated with a maximum distance between release and recapture of 200 m. Charlwood [62] observed such limited dispersal for *Anopheles funestus* in Mozambique using a classical MRR technique. In the context of the SIT, sterile males should thus be released in a grid pattern with 200 m between release points.

Although the participation of sterilized *An. arabiensis* in swarms has been demonstrated here, their competitiveness in achieving successful copulation in the field is not yet proven. One investigation in *An. culicifacies* showed that sterile males that rested and swarmed concomitantly with wild males were non-competitive in nature even though they were equally competitive in laboratory experiments [75]. However, according to Helinski *et al.* [50], ‘wild’ *An. arabiensis* females did not discriminate against ‘released’ sterile males from recently colonized lines in field cage experiments in Sudan. In future experiments, captures of paired mosquitoes leaving swarms *in copula* to check for the presence of sterile males in mating pairs would provide further evidence of their competitiveness. Released sterile males could also be marked with a stable isotope, allowing wild females to be trapped and analysed for the presence of the isotope transfered through mating with a sterile male [76, 77]. Moreover, to verify if the swarms are really attractive to the released males, the use of barrier screens around the release point [78] could give us the confirmation that dispersal of the released males were not random but biased towards the direction of the swarms.
